# Expression of miRNAs in turkey muscle satellite cells and differential response to thermal challenge

**DOI:** 10.3389/fphys.2023.1293264

**Published:** 2023-11-23

**Authors:** Kent M. Reed, Kristelle M. Mendoza, Thomas Kono, Ashley A. Powell, Gale M. Strasburg, Sandra G. Velleman

**Affiliations:** ^1^ Department of Veterinary and Biomedical Sciences, University of Minnesota, St. Paul, MN, United States; ^2^ Minnesota Supercomputing Institute, University of Minnesota, Minneapolis, MN, United States; ^3^ Department of Food Science and Human Nutrition, Michigan State University, East Lansing, MI, United States; ^4^ Department of Animal Sciences, The Ohio State University, Wooster, OH, United States

**Keywords:** microRNA, satellite cell, skeletal muscle, growth selection, turkey

## Abstract

Thermal stress alters the transcriptome and subsequent tissue physiology of poultry; thus, it can negatively impact poultry production through reduced meat quality, egg production, and health and wellbeing. The modulation of gene expression is critical to embryonic development and cell proliferation, and growing evidence suggests the role of non-coding RNAs (RNA:RNA interaction) in response to thermal stress in animals. MicroRNAs (miRNAs) comprise a class of small regulatory RNAs that modulate gene expression through posttranscriptional interactions and regulate mRNAs, potentially altering numerous cellular processes. This study was designed to identify and characterize the differential expression of miRNAs in satellite cells (SCs) from the turkey *pectoralis major* muscle and predict important miRNA:mRNA interactions in these developing SCs under a thermal challenge. Small RNA sequencing was performed on RNA libraries prepared from SCs cultured from 1-week-old male Nicholas commercial turkeys (NCTs) and non-selected Randombred Control Line 2 turkeys during proliferation and differentiation at the control temperature (38°C) or under a thermal challenge (33°C or 43°C). A total of 353 miRNAs (161 known and 192 novel) were detected across the sequenced libraries. Expression analysis found fewer differentially expressed miRNAs in the SCs of NCT birds, suggesting that the miRNA response to heat stress has been altered in birds selected for their modern commercial growth traits. Differentially expressed miRNAs, including those with described roles in muscle development, were detected both among temperature treatments and between genetic lines. A prominent differential expression of miR-206 was found in proliferating turkey SCs with a significant response to thermal challenges in both lines. In differentiating SCs, isoforms of miR-1 had significant differential responses, with the expression of miR-206 being mainly affected only by cold treatment. Target gene predictions and Gene Ontology analysis suggest that the differential expression of miRNAs during thermal stress could significantly affect cellular proliferation and differentiation.

## Introduction

Thermal stress can have a negative impact on poultry production. Both heat and cold stress have been shown to reduce meat quality, egg production, and wellbeing, including the overall health and quality of life in production birds (Henrikson et al., 2018; Barnes et al., 2019; Patael et al., 2019; Ouchi et al., 2021). Previous studies have shown that thermal challenges can systemically create changes in live birds at the transcriptomic and physiological levels, both in specific tissues, including the important food quality tissues and muscles (Reed et al., 2017a; Reed et al., 2017b; Al-Zghoul et al., 2019; Barnes et al., 2019; Xu et al., 2021a; Reed et al., 2022a; Reed et al., 2022b). Some of these changes are tissue-specific shifts in, for example, lipid synthesis and degradation pathways and the upregulation of protein degradation mRNAs and other stress response pathways (Barnes et al., 2019; Xu et al., 2021b). Comparisons of muscles from diverse genetic lines of poultry, specifically breast muscle (*pectoralis major*) stem cells (satellite cells, SCs), have shown differences in their response to thermal challenges (Wilson et al., 1975; Li et al., 2018; Xu et al., 2021a; Xu et al., 2021b). As self-renewing mesenchymal cells, SCs enable muscle hypertrophy, maintenance, and damage repair. Avian SCs are highly active in the early post-hatch period (Halevy et al., 2000; Mozdziak et al., 2002), and their activity can be altered by environmental stimuli with potential long-lasting effects on skeletal muscle growth (Piestun et al., 2013; Loyau et al., 2014). A better understanding of such responses could allow for targeted selection in breeding for creating more resilient birds.

Growing evidence suggests the role of non-coding RNAs, such as microRNAs (miRNAs), in the regulation of muscle growth and development and their response to thermal stress in animals (Andreote et al., 2014; Fu et al., 2018; Nawab et al., 2018; Sengar et al., 2018; Lang et al., 2019; Raza et al., 2021). MicroRNAs are 18–25 nt single-stranded RNAs that are thought to function primarily in posttranscriptional gene silencing by base pairing with target mRNAs, leading to destabilization, mRNA cleavage, or translational repression (Saliminejad et al., 2019). Gene silencing mediated by miRNAs plays an important role in animal development and disease (Kloosterman and Plasterk, 2006), with tissue-specific expressions being common in vertebrate development (Wienholds et al., 2005; Ason et al., 2006). Several studies have examined miRNA involvement in the skeletal muscle of poultry (Li et al., 2011; Andreote et al., 2014; Harding and Velleman, 2016; Velleman and Harding, 2017; Jebessa et al., 2018). Studies in chicken have shown that some miRNAs that are commonly differentially expressed in human muscle disorders are also differentially expressed in chicken muscle-development disorders (Shu et al., 2021). Other studies on chicken breast muscles have shown that miRNAs appear to be important during muscle development and the deposition of intramuscular fat, both of which can impact the final meat quality (Fu et al., 2018; Liu et al., 2021).

Little is known about miRNA expression and function in turkeys. A previous work by our group examined the role of three miRNAs (miR-16, miR-24, and miR-128) in the expression of genes essential to satellite cell function in turkeys. The inhibition of these miRNAs differentially affected the expression of syndecan-4, glypican-1, and myogenic regulatory factors, myogenic differentiation 1 (*myoD*) and myogenin (*MYOG*) (Harding and Velleman, 2016). Two of the miRNAs (miR-24 and miR-128) also played a role in myogenic satellite cell migration (Velleman and Harding, 2017). Further investigation of the miRNA expression and their functional interactions with mRNAs is needed to create a more complete picture of muscle development in production turkeys, particularly in regards to thermal stress. A critical initial step in identifying miRNA:mRNA target interactions is through miRNA characterization and computational prediction.

We have previously observed statistically significant differences in the gene expression (mRNA) of turkey *p. major* muscle SCs between growth-selected and non-selected birds and in response to thermal challenges (Reed et al., 2017a; Reed et al., 2017b; Reed et al., 2022a; Reed et al., 2022b). The current study was designed to identify miRNAs expressed in *p. major* muscle SCs, to identify promising candidates for further investigation, to characterize the differential expression of miRNAs, and to predict important miRNA:mRNA interactions in developing turkey skeletal muscle SCs. The experimental design of this study is novel, showing that the use of muscle satellite cells allows the delineation of their contribution independently from other cell types in the muscle tissue, whereby these mechanisms can be more clearly linked to satellite cell function. We hypothesized that the expression of miRNAs in turkey muscle SCs would be significantly altered by thermal challenges and would vary in cells from commercial growth-selected birds compared to non-selected birds.

## Materials and methods

RNA for this study was obtained from cultured SCs previously isolated from the *p. major* muscles of 1-week-old male Nicholas commercial turkeys (NCTs) and Randombred Control Line 2 (RBC2, representing commercial turkeys of 1966) turkeys. RBC2 turkeys were initiated in 1966 and maintained at the Poultry Research Center of The Ohio State University, Wooster, OH, without the conscious selection of any trait and were used as an important control in studies of select lines (Nestor et al., 1969). NCTs are modern meat-type turkeys obtained from Nicholas turkeys (Aviagen Group, Lewisburg, WV).

Pooled turkey SCs were cultured as described by Reed et al. (2017a) and Reed et al. (2022a). In brief, the SCs from both lines were plated in 0.1% gelatin (Sigma-Aldrich, St. Louis, MO)-coated 24-well plates (Greiner Bio-One, Monroe, NC) with 15,000 cells per well in Dulbecco’s Modified Eagle’s Medium (DMEM, Sigma-Aldrich) plating medium containing 10% chicken serum (Gemini Bio-Products, West Sacramento, CA), 5% horse serum (Gemini Bio-Products), 1% antibiotics–antimycotics (Gemini Bio-Products), and 0.1% gentamicin (Gemini Bio-Products). Satellite cells were incubated in a 95% air/5% CO_2_ incubator (Thermo Fisher Scientific, Waltham, MA) at 38°C for 24 h. After 24 h of attachment, the plating medium was replaced with McCoy’s 5A (Sigma-Aldrich) growth medium containing 10% chicken serum (Gemini Bio-Products, West Sacramento, CA), 5% horse serum (Gemini Bio-Products), 1% antibiotics–antimycotics (Gemini Bio-Products), and 0.1% gentamicin (Gemini Bio-Products) for 72 h. The growth medium was refreshed every 24 h for 72 h. After 72 h of proliferation, the growth medium was replaced with a DMEM differentiation medium containing 3% horse serum, 1% antibiotics–antimycotics, 0.1% gentamicin, 0.1% gelatin, and 1 mg/mL bovine serum albumin (BSA, Sigma-Aldrich) for 72 h of differentiation. The differentiation medium was changed every 24 h for 72 h.

### Proliferation experiment

After 24 h in the plating medium, the cells fed the growth medium and within the treatment were replicate-cultured at an experimental temperature (33°, 38°, or 43°C) for 72 h with the medium being replaced every 24 h. The control temperature of 38°C is the approximate temperature measured in newly hatched poults (38.0°C–38.5°C, G. Strasburg, unpublished data), and heat and cold treatments (43°C and 33°C, respectively) deviate from the approximate body temperature of mature turkeys (41.5°C). These temperatures have been shown to produce significant effects on satellite cell proliferation (Clark et al., 2016; Xu et al., 2021a). At harvest, SCs were collected using RNAzol RT (Sigma-Aldrich) and stored at −80°C until RNA isolation.

### Differentiation experiment

For differentiation, the cells were cultured as previously described by Reed et al. (2017b) and Reed et al. (2022b). The cells within the treatment were replicate-plated at 38°C (control) or at one of the challenge temperatures (33°C or 43°C), and the medium was changed every 24 h for the 72 h of differentiation. The cells were harvested as mentioned previously.

### RNA isolation and sequencing

Total RNA was isolated from each sample by RNAzol RT (Sigma-Aldrich) extraction, DNase-treated (TURBO DNA-free TM Kit, Ambion, Inc.), and stored at −80°C. The initial RNA concentration and quality were assessed by spectrophotometry (NanoDrop 1000), and the samples were submitted for library preparation and sequencing at the University of Minnesota Genomics Center. Each sample was further quantified by the RiboGreen assay (Invitrogen Corp.) using the 2100 Bioanalyzer (Agilent Technologies). Due to poor cell growth during proliferation at 33°C, the RNA quantity for the RBC2 treatment group was insufficient, and this group was excluded from further analysis. For each of the remaining treatment groups, the replicate samples were prepared for sequencing (two biological replicates per treatment group). Indexed libraries (*n* = 22) were constructed using the Takara Bio smRNA Library Preparation Kit and sizes selected for approximately 170 bp inserts. The libraries were multiplexed and sequenced on the NovaSeq SP platform using v1.5 chemistry (Illumina, Inc.) to produce 51-bp paired-end reads (data accessioned as part of the NCBI SRA BioProject PRJNA842679).

### Illumina sequence data handling

Illumina sequencing reads were screened for low-quality bases and adapter contamination with FastQC 0.11.9 (https://www.bioinformatics.babraham.ac.uk/projects/fastqc/). Per-library FastQC reports were aggregated into a joint report for easy browsing using MultiQC 1.13 (Ewels et al., 2016). For each sequenced library, the “forward” (R1) read was used for downstream analyses. Sequencing adapters were removed from the reads using cutadapt 4.2 (Martin, 2011). The first three bases were additionally removed during trimming to remove non-biological bases added during library preparation. Reads were removed if their lengths were shorter than 15 nt after trimming. Trimmed reads were then cleaned of ribosomal sequences using BBDuk 39.01 (https://sourceforge.net/projects/bbmap/). The reference sequences used for ribosomal depletion were large subunit and small subunit ribosomal sequences retrieved from SILVA release 132 (Quast et al., 2013). Reads were removed if they had an exact match of at least 15 nt to one of the reference sequences from SILVA. Reads that were trimmed of adapters and depleted of ribosomal sequences were used for downstream analyses.

### miRNA prediction

Cleaned reads from all libraries were combined into a single file for the prediction of novel miRNAs against the turkey genome. The turkey genome assembly (GCA_943295565.1) was prepared for mapping using Bowtie 1.3.1 (Langmead et al., 2009). Characterized mature miRNAs from chicken (*Gallus gallus*) were downloaded from miRBase release 22.1 to use as previously known miRNAs. Novel miRNAs were predicted in the combined sequencing libraries using miRDeep2 0.1.2 (Friedländer et al., 2012), and miRNA sequences were retained if their miRDeep2 score was >0.

### miRNA expression profiling

Cleaned reads from each library were separately mapped to the turkey genome assembly using Bowtie 1.3.1 (Langmead et al., 2009). The options used were “-n 0 -e 80 -l 15 -m 5 --best--strata” to recreate the same parameters that were used for miRNA discovery using miRDeep2. SAM files were converted to BAM files using SAMtools 1.14 (Danecek et al., 2021). Processing of alignment files was performed in parallel with GNU parallel version 20210822 (Tange, 2018). miRNA regions identified using miRDeep2 were converted to SAF files for expression quantification. miRNA expression was quantified using “featureCounts” version 2.0.3 (Liao et al., 2014), requiring a minimum mapping quality of 10 for a read to be counted. To identify differentially expressed miRNAs (DEMs), expression values were first normalized by the library size and multiplied by a factor of 1 × 10^6^, corresponding to counts per million (CPM) mapped miRNA reads, where the library size is the total number of reads mapped to miRNA precursors. The counts matrix from featureCounts was analyzed using the “edgeR” package (Robinson et al., 2010) in the R statistical computing environment, version 4.2.2 (R Core Team, 2022). miRNAs with a low expression were filtered by removing those that did not have at least three assigned reads in at least two libraries. Global patterns in miRNA expression were assessed with principal component analysis using the prcomp function in R. Variance partitioning analyses were conducted using the “variancePartition” package (Hoffman and Schadt, 2016) in R, estimating the contributions of the incubation temperature, genotype, and an interaction between the incubation temperature and the genotypic variance in miRNA expression. Differential expression analyses were carried out with the quasi-likelihood F-test using edgeR (Chen et al., 2016). Differences were evaluated for the fold change (log_2_FC) and were considered significant at *p* <0.05. BioVenn (Hulsen et al. (2008) was used to create Venn diagrams.

### miRNA target prediction

Potential miRNA target genes were initially predicted using TargetScan 8.0 (McGeary et al., 2019) and miRDB (Chen and Wang, 2020) using the chicken miRNA database. The given sequence differences between turkey and chicken genomes and potential binding sites were subsequently computationally predicted using miRanda v3.3a (Enright et al., 2003). Although the trio-based turkey genome assembly (GCA_943295565.1) offers improved coverage and assembly quality, at the time of this study, it had not been annotated in either the Ensembl or NCBI databases. Therefore, to identify the genes associated with the predicted turkey miRNAs, we used the reference UMD-5.1 assembly to align the miRDeep2-predicted consensus precursor sequences via BLAST. Gene targets in turkey were predicted by aligning the miRNA sequences against all RNA transcripts in the annotated UMD-5.1 genome build (NCBI annotation 104) with position-weighted scoring, an alignment score of >150, and |energy-kcal/mol| >7.0. Enrichment tests for target genes were performed using the PANTHER Overrepresentation Test [GO Consortium release 20150430 (Mi et al., 2013); http://geneontology.org/]. GO analysis utilized the chicken (*G. gallus*) reference gene list with ∼66% of turkey loci (Annotation 105) having ID homologs. The differences were considered significant at *p* <0.05.

### Workflow availability

All scripts needed to recreate the analyses described previously are available in a GitHub repository at https://github.com/TomJKono/Turkey_MSC_miRNA.

## Results

### Small RNA sequencing

**FIGURE 1:**
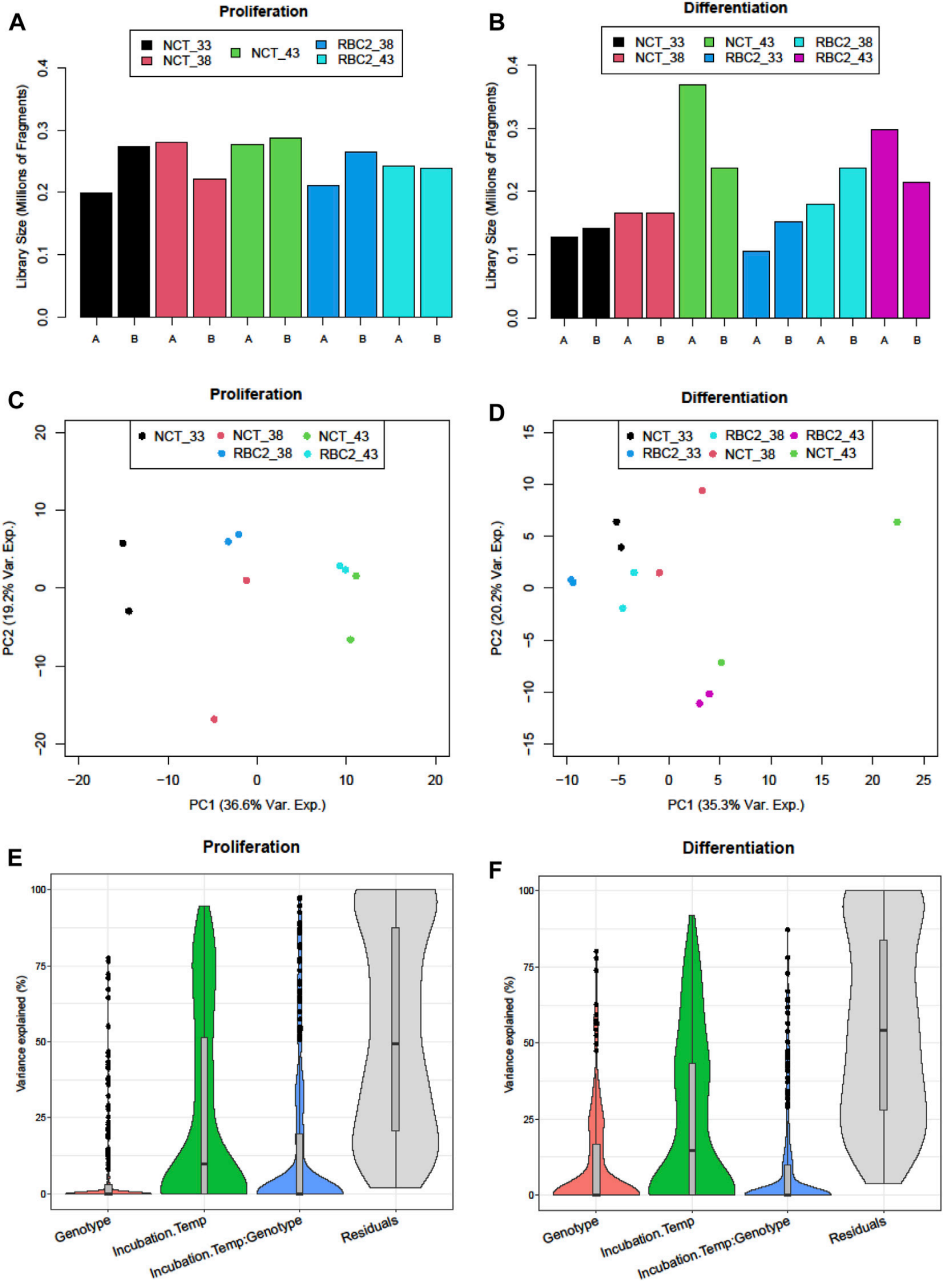
Distribution of sequencing reads (millions of fragments) in sequencing libraries of treatment groups of **(A)** proliferating and **(B)** differentiating muscle satellite cells. PCA plots of normalized read counts for **(C)** proliferating and **(D)** differentiating satellite cells. Sample-to-sample distances (within and between treatments) are illustrated for each treatment sample on the first two principal components. The samples are plotted according to the treatment. The distribution of sample variance by the treatment factor: genotype (line), temperature, interaction, and residual for **(E)** proliferating and **(F)** differentiating satellite cells.

The results for the sequencing of the small RNAs are summarized in Table 1. The number of PF (passing filter) clusters averaged 19.52M reads encompassing an average 1,991 Mb per library. The read quality was consistently high with an average mean Q score of 32.4. Library sizes from the proliferation experiment were very similar (Figure 1A) and were averaged higher than those from the differentiation experiment where the numbers of reads were more variable among libraries (Figure 1B). Here, the number of reads was the lowest in the 33°C treatment replicates and the highest in the 43°C replicates.

**TABLE 1:** Summary of RNA-seq data used for miRNA discovery and expression analyses^a^.

Experiment	Line	Temperature (°C)	Replicate	PF cluster	Yield (Mb)	% ≥Q30 bases	Mean Q score
Proliferation	NCT	33	A	16,185,368	1,651	77.54	32.40
	NCT	33	B	18,663,090	1,904	77.35	32.37
	NCT	38	A	18,513,867	1,888	77.07	32.31
	NCT	38	B	15,089,576	1,539	77.49	32.38
	NCT	43	A	19,911,181	2,031	77.38	32.35
	NCT	43	B	20,772,409	2,119	77.73	32.44
	RBC2	38	A	15,555,080	1,587	77.08	32.32
	RBC2	38	B	18,487,011	1,886	77.49	32.39
	RBC2	43	A	19,835,488	2,023	77.73	32.44
	RBC2	43	B	19,131,708	1,951	77.34	32.37
Differentiation	NCT	33	A	21,728,024	2,216	77.68	32.41
	NCT	33	B	21,552,098	2,198	77.84	32.46
	NCT	38	A	20,274,703	2,068	77.87	32.47
	NCT	38	B	20,879,610	2,130	77.76	32.43
	NCT	43	A	30,452,556	3,106	78.65	32.63
	NCT	43	B	16,756,472	1,709	77.48	32.38
	RBC2	33	A	16,405,230	1,673	77.48	32.36
	RBC2	33	B	23,417,907	2,389	77.88	32.46
	RBC2	38	A	16,314,326	1,664	77.80	32.44
	RBC2	38	B	22,244,407	2,269	77.94	32.47
	RBC2	43	A	21,406,067	2,183	77.65	32.42
	RBC2	43	B	15,893,508	1,621	77.87	32.46

^a^ For each library, the total number of PFs, clusters (the number of reads passing filter in millions per lane), yield (Mbases), percentage of bases with the quality score (Q) ≥30, and mean Q score are given.

### Identification and the expression of conserved and novel miRNAs

Clean reads obtained from all sequencing libraries in both experiments were used for the detection of expressed miRNAs in the SCs using miRDeep2. The performance of miRDeep2 in the detection of known miRNAs (those identified based on the sequence comparison of their miRNA precursors with the miRBase dataset of *G. gallus*) and novel miRNAs is presented in Supplementary Table S1. In this study, a total of 353 miRNAs (161 known and 192 novel) were detected. The expression of putative novel miRNAs was lower than that of the known miRNAs.

Novel miRNAs were considered high-confidence if both the putative mature and star miRNAs (miRNA corresponding to the other side of the hairpin) reported by miRDeep2 were detected in at least two independent samples, having the exact same 5′- and 3′-ends and allowing no mismatches. The cutoff values for confidence are somewhat arbitrary for novel miRNA predictions, but some studies suggest that a miRDeep score >1, significant RNAfold *p*-value, and mature reads >10 can be used as minimum values. Based on these criteria, 118 of the 192 detected novel miRNAs (61.4%) were considered high-confidence. In addition, sequencing reads were found to map to various miRBase gga-miRNAs that were not included within the “known” category. These “known miRNAs not detected using miRDeep2” were observed due to unusual precursor structures that do not fit the assumed biogenesis model of miRDeep2. The gga-miRNAs classified as “not detected” included 14 gga-miRNAs with mapped read counts >100 among the libraries (Supplementary Date Sheet S1). Although these were not included in subsequent analyses, the expression of three of these (gga-let-7b [140,731 mapped reads], gga-let-7l-2 [19,029], and gga-mir-210a [15,620]) may warrant further investigation. Using the UMD-5.1 genome assembly, 150 known and 130 novel miRNAs were uniquely mapped to the turkey genome (Supplementary File 1). Based on the NCBI UMD-5.1 annotation (v104), 181 (51.3%) of the precursor sequences (92 and 89 of the known and novel miRNAs, respectively) occurred within annotated genes.

### Thermal challenge of proliferating satellite cells

**FIGURE 2:**
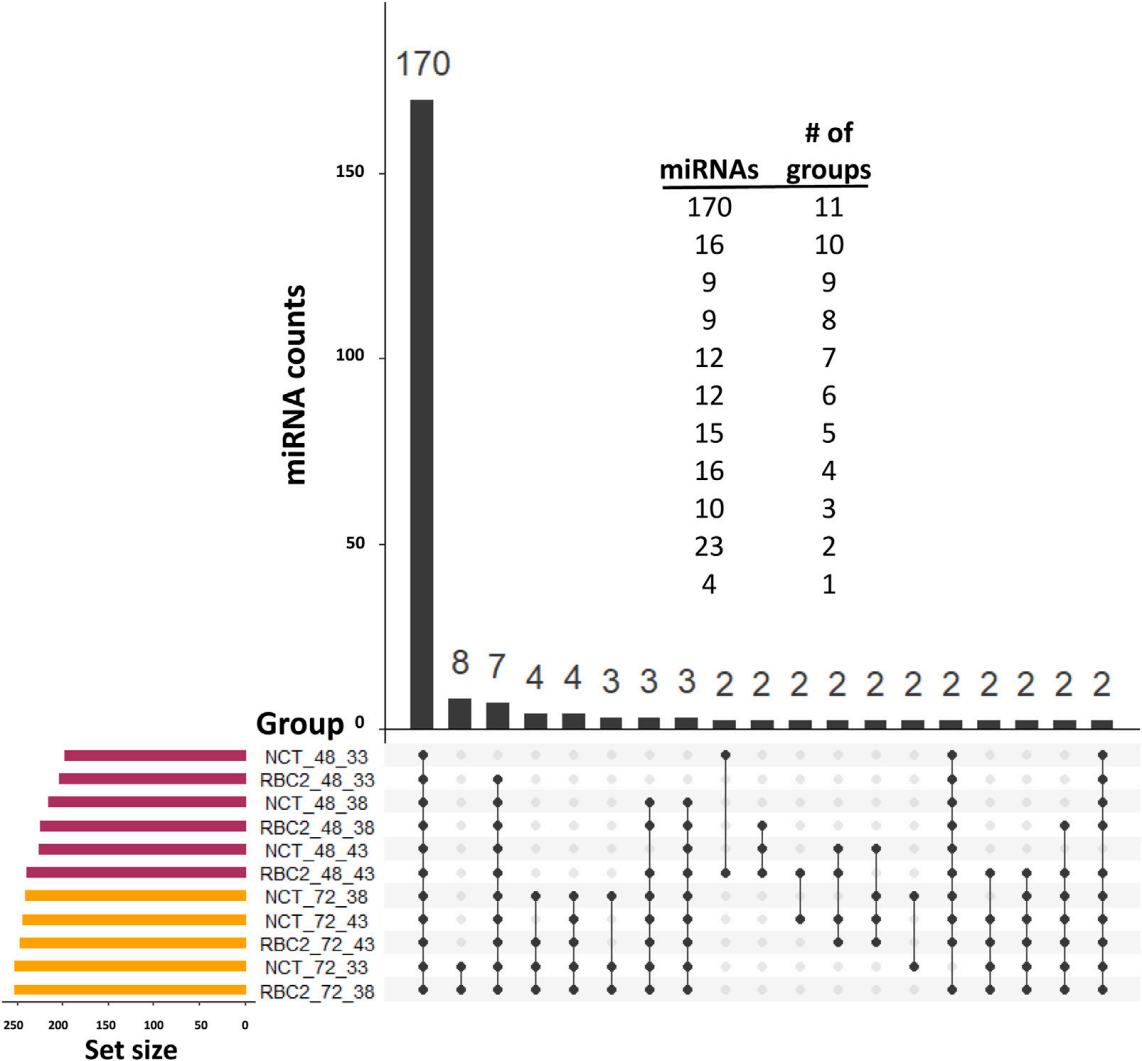
UpSet plot (Conway et al., 2017) of the expressed miRNAs. For inclusion, miRNAs must first have at least three assigned reads in at least two libraries and a treatment group average number of reads of >2.0. The horizontal bars on the left indicate the number of miRNAs expressed in each treatment. Individual points in the matrix represent miRNAs specific to each treatment, and the lines between points represent the miRNAs common to different groups. We excluded 70 single miRNAs that individually had unique group distributions. The vertical bars above indicate the number of miRNAs specific to or common to different treatments. The distribution of miRNAs by the number of treatment groups in which they were included is given above.

Distributions of the expressed miRNAs are summarized in Figure 2. In the observed expression of 294 miRNAs, 170 were common in all treatments in both experiments (proliferation and differentiation). The expression of 39 miRNAs was low (average of 7.5 reads/treatment) and was limited to proliferating SCs. Only four miRNAs were uniquely expressed in single-treatment groups in the proliferation experiment (one each in the NCT 33°C, NCT 38°C, RBC2 38°C, and RBC2 43°C treatments), and all of them were novel miRNAs with a low expression (average of 2.6 reads/treatment).

Variation in the expression among treatment groups was visualized by the principal component analysis (PCA). In the proliferation experiment, treatment groups clustered distinctly along the first principal component (PCA1) (Figure 1C) with replicate treatment pairs occurring as nearest neighbors within the PCA space. The greatest within-treatment separation was seen for the NCT samples in the control (38°C) treatment along the PCA2. Variance partitioning was used to estimate the contributions of the incubation temperature, genotype, and temperature × genotype interaction and found that the incubation temperature explained a greater proportion of the variation than genetic background (Figure 1E).

#### Identification of differentially expressed miRNAs

Normalization by the library size resulted in a counts matrix of 271 miRNAs (151 known and 120 novel) for analyses with EdgeR (Supplementary Table S2). With heat treatment (43°C), only a single DEM (miR-206) was identified in NCT SCs in comparison to the control temperature (38°C). The expression of this miRNA was significantly elevated by heat treatment (log_2_FC = 3.74). In contrast, heat treatment of RBC2 SCs had a greater effect on the miRNA expression where 73 DEMs (44 known and 29 novel miRNAs) were identified in comparison to the control temperature (38°C) (Supplementary Table S3). Of these 73 DEMs, 34 were upregulated and 39 downregulated by heat treatment. Twenty nine of the 73 DEMs had |log_2_FC| >1.0, and 12 had |log_2_FC| >2.0. The greatest upregulation was observed for miR-206 (log_2_FC = 4.41), miR-N145 (3.69), and miR-N34 (2.75). The greatest downregulation was observed for the novel miRNAs miR-N77 (log_2_FC = −4.0), miR-N23 (−3.52), miR-N54 (−3.31), and miR-N82 (−2.96). Libraries for SCs proliferating at 33°C were only sequenced for the NCT line, and no DEMs were identified in comparison to these with the control temperature.

**FIGURE 3:**
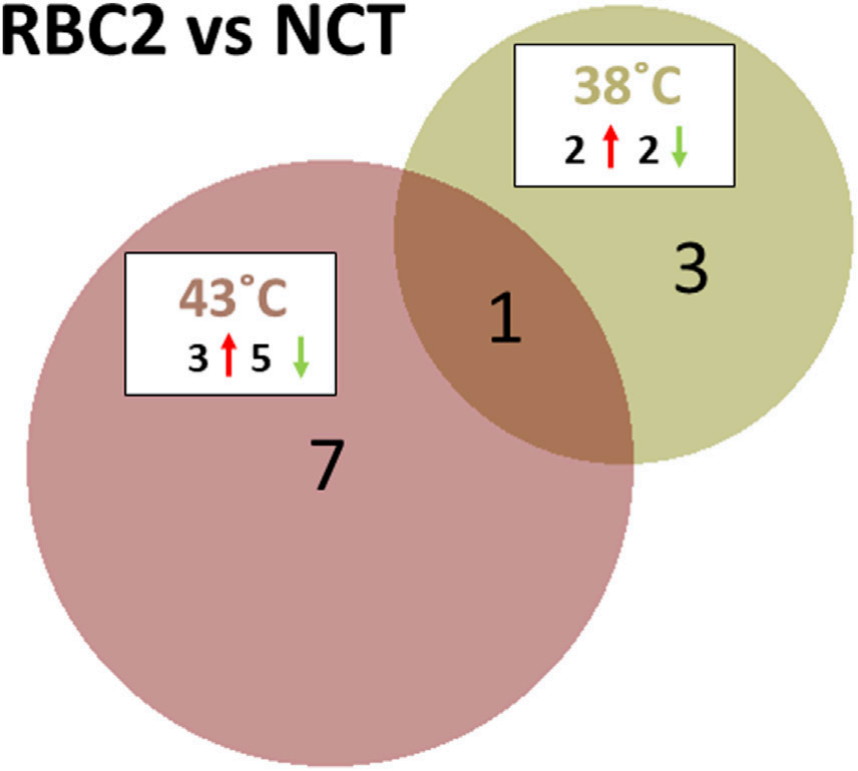
Distribution of DEMs during the proliferation of cultured turkey *p. major* SCs. For each temperature comparison, the DEMs with FDR *p*-value <0.05 that were shared or unique to each line (RBC2 and NCT) are indicated in the Venn diagram. The circle size is proportional to the number of DEMs.

The response of the two turkey lines was dramatically different at the control and heat treatment temperatures. At the control temperature (38°C), four miRNAs were found to be differentially expressed between the SCs of commercial birds (NCT) and the RBC2 line (Figure 3; Supplementary Table S3). These included the known miRNAs, miR-206 and miR-184-5p, where the expression was lower in RBC2 SCs (log_2_FC = −2.29 and −1.58, respectively). The novel DEMs, miR-N96 and miR-N173, had higher expressions of RBC2 SCs (log_2_FC = 1.34 and 2.57, respectively).

At the elevated temperature (43°C), line comparison of RBC2 and NCT SCs found eight DEMs (Figure 3; Supplementary Table S3). These included six known miRNAs, miR-129-5p, miR-146b-5p, miR-206, miR-1416-5p, and two isoforms of miR-2954. Log_2_FC for these DEGs was generally low (log_2_FC = −0.91 to 0.84), with the exception of miR-206 (−1.63), where the expression in RBC2 cells was significantly lower compared to NCT SCs, similar to those seen at the control temperature. The two novel DEMs, miR-N154 and miR-N185, were both upregulated in RBC2 cells compared to NCT (log_2_FC = 0.78 and 1.39, respectively).

### Thermal challenge of differentiating SCs

A total of 255 predicted miRNAs were observed in SCs during differentiation, with only eight being uniquely expressed in the differentiating cells (Figure 2). The unique transcripts were a mix of known (six) and novel (two) miRNAs with a low average expression (3.7 reads/treatment group). Treatment groups clustered distinctly within the first two principal components (Figure 1D) and replicate samples generally clustered together with the exception of NCT 43°C samples, which had the largest separation within the PCA space. Variance partitioning found the incubation temperature to explain the largest proportion of the variation (Figure 1F), and the genotype had a greater variance component in differentiating SCs when compared to proliferating SCs.

#### Differentially expressed miRNAs following heat treatment

**FIGURE 4:**
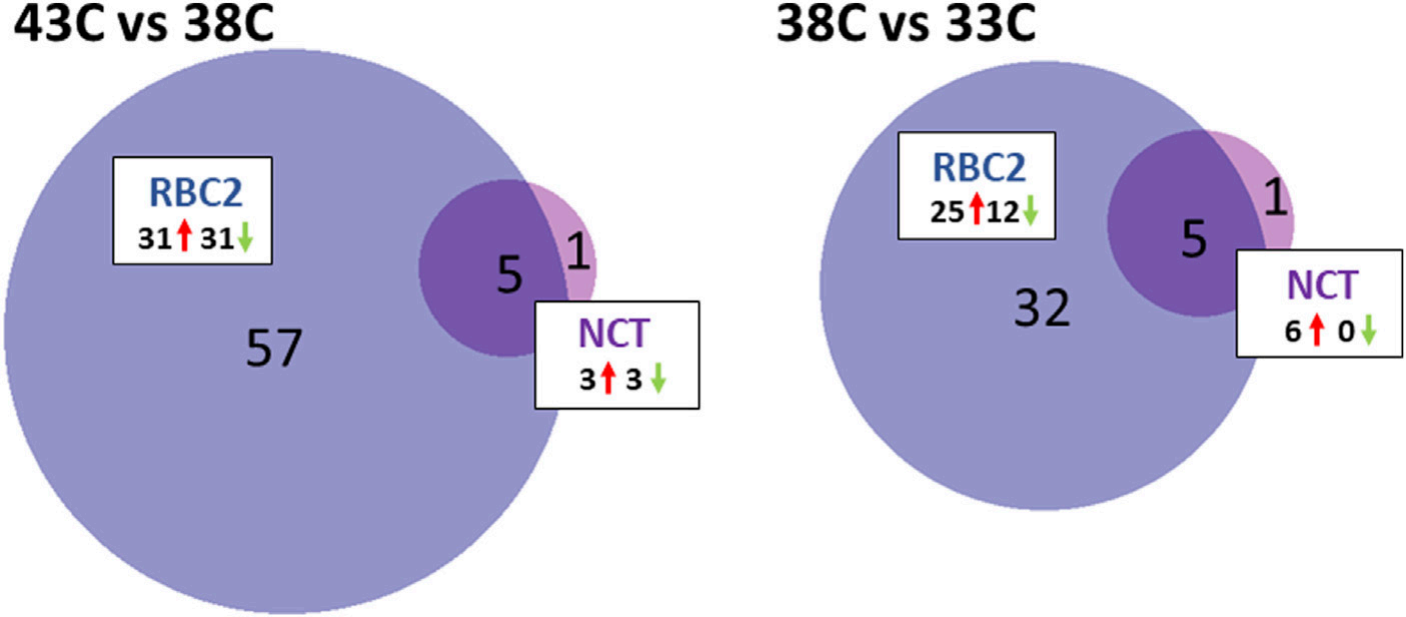
Distribution of DEMs during the differentiation of cultured turkey *p. major* SCs. For each temperature comparison, the DEMs with FDR *p*-value <0.05 that were shared or unique to each line (RBC2 and NCT) are indicated in the Venn diagram. The circle size is proportional to the number of DEMs.

The counts matrix for the differentiation experiment included 213 miRNAs (148 known and 65 novel) (Supplementary Table S4). Several DEMs were found in within-line comparisons between the 43°C and 38°C treatments (Figure 4, Supplementary Table S5). In NCT SCs, six DEMs were identified including the known miR-1559-5p and five novel miRNAs (miR-N29, miR-N105, miR-N140, miR-N157, and miR-N183). Directional regulation was split, with three miRNAs (miR-N105, miR-N140, and miR-N183) being upregulated in NCT SCs (log_2_FC = 6.22, 2.62, and 2.12, respectively) at 43°C and three (miR-N29, miR-1559-5p, and miR-N157) being downregulated (log_2_FC = −5.02, −2.44, and −2.19, respectively).

The response to heat treatment was more significant in RBC2 SCs than that in NCTs. In the RBC2 within-line comparison (43°C vs. 38°C), 62 DEMs were identified (Figure 4; Supplementary Table S5), including 39 known and 23 novel miRNAs, with 31 being upregulated and 31 downregulated with heat treatment. The log_2_FC of these DEMs ranged from 6.98 to −3.19 with 35 having |log_2_FC| >1.0 and 13 with |log_2_FC| >2.0. The greatest upregulation was observed for miR-205b (log_2_FC = 6.98) and miR-N30 (4.17), and the largest downregulation was observed for miR-N54 (log_2_FC = −3.19) and miR-460b-5p (−2.25).

Five DEMs were shared between the NCT and RBC2, 43°C vs. 38°C, comparisons. The expression of miR-N105, miR-N140, and miR-N183 was significantly higher in heat-treated cells than that in the controls in both lines. In contrast, miR-N157 and miR-1559-5p were significantly downregulated in heat-treated cells compared to controls.

DE analysis found no significant differences in miRNA expression between RBC2 and NCT SCs at either the control temperature (38°C) or heat treatment (43°C).

#### Differentially expressed miRNAs following cold treatment

A within-line comparison found six DEMs in the NCT SCs being incubated at 33°C relative to controls (38°C). These included three isoforms of miR-1 and three novel miRNAs (miR-N183, miR-N140, and miR-N30) (Figure 4; Supplementary Table S5). Each of the DEMs showed higher levels of expression at 38°C compared to 33°C with average log_2_FC >3.0.

As seen for heat treatment, the proliferating RBC2 SCs showed a greater response to cold treatment than the NCT SCs (Figure 4; Supplementary Table S5). The comparison of the 33°C-treated cells to the control identified 37 DEMs, including 24 known and 13 novel miRNAs, with 25 being upregulated and 12 downregulated. Overall, log_2_FC ranged from 4.83 to −3.18 with 20 DEMs having |log_2_FC| >1.0 and eight with |log_2_FC| >2.0. The greatest fold change was observed for two predicted novel miRNAs (miR-N29 and miR-102; log_2_FC = 4.83 and 2.89, respectively), with a higher expression at 38°C. The novel miRNA, miR-N68, showed the greatest expression change at 33°C (log_2_FC = −3.18). Five DEMs were shared between NCT and RBC2, 33°C vs. 38°C, comparisons. In SCs, from both lines, the expression of miR-1a-1-5p, miR-1a-2-5p, miR-1b-5p, miR-N140, and miR-N183 was significantly lower in cold-treated cells compared to controls.

Significant differences in miRNA expression were observed between the lines with cold treatment (33°C), and 33 miRNAs were differentially expressed between RBC2 and NCT SCs (Supplementary Table S5). This group of DEMs was primarily comprised of known miRNAs (29) but with a relatively low overall fold change (average log_2_FC = 0.106). The greatest upregulation was observed for miR-N72 and two isoforms of miR-1677-5p (log_2_FC = 2.69, 1.87, and 1.87, respectively). A significant downregulation was the greatest for miR-N63, miR-206, and miR-1416-5p (log_2_FC = −1.81, −1.25, and −1.11, respectively).

### miRNA target predictions

Target predictions used sequences of each of the DEMs (|log_2_FC| >1.0) to query the annotated transcript sequences in the turkey genome for potential miRNA target sites.

#### Genes targeted by DEMs in proliferating cells

Target predictions for the four DEMs identified in the RBC2 vs. NCT (38°C) line comparison averaged 1,515 target sites within 751 genes. Genes containing predicted sites with the highest alignment score are summarized in Supplementary Table S6. For the downregulated DEMs (miR-184 and miR-206), these included genes such as *ATG16L1* (autophagy-related 16-like 1), *KLHL7* (kelch-like family member 7), *KCNT1* (potassium- and sodium-activated channel subfamily T member 1), *MILR1* (mast cell immunoglobulin-like receptor 1), *HLCS* (holocarboxylase synthetase), *CPEB1* (cytoplasmic polyadenylation element-binding protein 1), and an uncharacterized locus (*LOC104914368*). The downregulation of the expression of these miRNAs could predictably increase the translation of these target genes. GO analysis of the suite of predicted targets of the two downregulated miRNAs (miR-184 and miR-206) showed significant enrichment (1.42×; *p* = 1.52E-02) for localization (GO:0051179). For the upregulated novel DEMs (miR-N96 and miR-N173), target genes included *LOC100541175* (keratin type-I cytoskeletal 13-like), *PDE6C* (phosphodiesterase 6C), *CRAT* (carnitine O-acetyltransferase), *EPHX1* (epoxide hydrolase 1), *EXD2* (exonuclease 3′–5′ domain-containing 2), and *USP47* (ubiquitin-specific peptidase 47). The upregulation of the expression of these miRNAs could predictably decrease the translation of these target genes. For these upregulated miRNA targets, GO molecular function terms for protein binding (GO:0005515; 1.29×; *p* = 4.05E-03) and ion binding (GO:0043167; 1.26×; *p* = 1.24E-02), and GO biological process terms for the apoptotic signaling pathway in response to DNA damage (GO:0008630; 5.71×; *p* = 2.24E-02) and cellular localization (GO:0051641; 1.51×; *p* = 1.04E-02) were significantly enriched.

In the heat-treated cells (43°C), only two of the eight DEMs had |log_2_FC| >1.0. These include miR-206 (downregulated in RBC2 relative to NCTs, −1.631) and miR-N185 (upregulated in RBC2, 1.392). Targets for miR-206 are as presented previously (Supplementary Table S6), and the top targets for miR-N185 included *HSPB7* (heat-shock protein family B (small) member 7) and *SCN2B* (sodium voltage-gated channel beta subunit 2). Given the increased number of DEMs identified in RBC2 cells under heat treatment (73), the number of potential miRNA interaction sites is significantly increased compared to those in NCT cells. Of the 73 DEMs, 29 had |log_2_FC| >1.0, and target site prediction identified nearly 2,500 predicted target sites within an average 1,065 genes per miRNA. Targets for the top 10 genes per miRNA are given in Supplementary Table S7.

GO analysis of the suite of predicted targets for miRNAs downregulated by heat treatment found the highest significant enrichment for the GO biological process term ubiquitin-dependent protein catabolic process (GO:0006511; 1.54×; *p* = 2.18E-02), protein catabolic process (GO:0030163; 1.49×; *p* = 1.00E-02), proteolysis involved in the protein catabolic process (GO:0051603; 1.48×; *p* = 4.65E-02), and cellular response to stress (GO:0033554; 1.47×; *p* = 3.42E-05) and for GO molecular function term identical protein binding (GO:0042802; 1.55×; *p* = 3.45E-04) and enzyme binding (GO:0019899; 1.41×; *p* = 1.13E-03). Analysis of targets for upregulated miRNAs found the highest significant enrichment for the GO biological process term regulation of cell differentiation (GO:0045595; 1.52×; *p* = 5.57E-03), regulation of the developmental process (GO:0050793; 1.48×; *p* = 3.47E-05), and cellular response to stress (GO:0033554; 1.46×; *p* = 2.67E-04) and for the GO molecular function term identical protein binding (GO:0042802; 1.5×; *p* = 1.10E-02).

#### Genes targeted by DEMs in differentiating cells

In the heat-treated cells, no DEMs were observed between the cell lines (NCT vs. RBC2), and fewer expression differences were observed within NCT lines when comparing the heat treatment group (43°C) to the control (38°C). In the NCT comparison, six DEMs had |log_2_FC| >1.0, with an average of 943 targets identified among an average of 548.3 genes (Supplementary Table S8). Among the downregulated DEMs (miR-1559-5p, miR-N29, and miR-N157), the top gene targets included *HNRNPH3* (heterogeneous nuclear ribonucleoprotein H3), *KIF9* (kinesin family member 9), *KAT14* (lysine acetyltransferase 14), *AGRN* (agrin), *BCL11B* (BAF chromatin remodeling complex subunit BCL11B), and *HACD1* (3-hydroxyacyl-CoA dehydratase 1). Predicted targets for these three miRNAs were downregulated by cold treatment and found significant enrichment for the GO biological process term regulation of the cellular process (1.27×; *p* = 5.87E-07). Among the upregulated DEMs (miR-N105, miR-N140, and miR-N183), the top gene targets included *PCSK7* (proprotein convertase subtilisin/kexin type 7), *COL18A1* (collagen type XVIII alpha 1 chain), *ZBTB17* (zinc finger and BTB domain-containing 17), *JPH2* (junctophilin 2), *CAMTA1* (calmodulin-binding transcription activator 1), *FGFRL1* (fibroblast growth factor receptor-like 1), and *TMEM132A* (transmembrane protein 132A). Biological processes of secretion (GO:0046903; 2.39×; *p* = 1.52E-06), organic acid transport (GO:0015849; 2.36×; *p* = 3.04E-05), carboxylic acid transport (GO:0046942; 2.30×; *p* = 5.24E-05), and secretion by cell (GO:0032940; 2.28×; *p* = 2.48E-05) showed the greatest fold enrichment.

Similar to the proliferating SCs, an increased number of DEMs (62) was observed in the differentiating RBC2 cells under heat treatment, significantly increasing the number of potential miRNA interaction sites as compared to the NCT cells. Of the 62 DEMs, 35 had |log_2_FC| >1.0, and target site prediction identified an average of 1888.4 predicted target sites within an average of 912.6 genes per miRNA. Targets for the top 10 genes for each miRNA are given in Supplementary Table S9. GO analysis of the 7,104 potential target genes for the 17 downregulated miRNAs (|log_2_FC| >1.0) found the greatest enrichment for the biological process term apoptotic signaling pathway (GO:0097190; 1.88×; *p* = 3.19E-04), multicellular organismal-level homeostasis (GO:0048871; 1.81×; *p* = 1.43E-04), and macroautophagy (GO:0016236; 1.81×; *p* = 6.45E-04). GO analysis of the 6,980 potential target genes for the 18 upregulated miRNAs (|log_2_FC| >1.0) found the biological process term cellular component disassembly (GO:0022411; 1.75×; *p* = 2.75E-04), extracellular matrix organization (GO:0030198; 1.69×; *p* = 7.02E-04), and programmed cell death (GO:0012501; 1.59×; *p* = 1.95E-05) were significantly overrepresented.

In cold-treated cells (33°C), target predictions for the eight DEMs (|log_2_FC| >1.0) identified in the line comparison (RBC2 vs. NCT) averaged 1837.7 target sites within 850 genes. Genes containing predicted target sites with the highest alignment score are summarized in Supplementary Table S10. For the downregulated DEMs (miR-206, miR-1416-5p, miR-2954 (two isoforms), and miR-N63), these included genes such as *MILR1* (mast cell immunoglobulin-like receptor 1), *CACNA1E* (calcium voltage-gated channel subunit alpha 1E), *PLEKHM2* (pleckstrin homology and RUN domain-containing M2), *HLCS* (holocarboxylase synthetase), *UTP18* (UTP18 small subunit processome component), *CPEB1* (cytoplasmic polyadenylation element-binding protein 1), *LMX1A* (LIM-homeobox transcription factor 1 alpha), *TMEM109* (transmembrane protein 109), *TYW5* (tRNA–yW synthesizing protein 5), *LOC104913138* (protein ABHD14B-like), and an uncharacterized locus (*LOC104914368*). The downregulation of the expression of these miRNAs could predictably increase the translation of these target genes. The overrepresentation test found significant enrichment for the molecular function protein homodimerization activity (GO:0042803; 2.11×; *p* = 4.79E-05). For the upregulated DEMs (miR-1677 (two isoforms) and miR-N72), the targets included genes such as *BSDC1* (BSD domain-containing 1), *AGTR1* (angiotensin II receptor type 1), *CKB* (creatine kinase B), *LOC104911408* (N-acetylneuraminate 9-O-acetyltransferase-like), *MYCL* (MYCL proto-oncogene), bHLH (transcription factor), and *KCNJ13* (potassium inwardly rectifying channel subfamily J member 13). Overrepresentation associated with the predicted target genes included the biological process negative regulation of the cellular process (GO:0048523; 1.42×; *p* = 5.12E-05).

As seen in the proliferating cells, NCT SCs showed that fewer miRNAs were significantly affected by cold treatment. In NCT cells, all six DEMs were upregulated and had |log_2_FC| >1.0, and target predictions for these miRNAs averaged 1,591.2 targets within an average of 759.8 genes (Supplementary Table S11). The highest target alignment scores for these upregulated miRNAs (miR-1, three isoforms: miR-N30, miR-N140, and miR-N183) included *FSD2* (fibronectin type III and SPRY domain containing 2), *CSRNP1* (cysteine and serine rich nuclear protein 1), *TMEM132A* (transmembrane protein 132A), and the uncharacterized *LOC104914368*. GO analysis implicates the regulation of blood circulation (GO:1903522; 2.72×; *p* = 8.61E-05) as an overrepresented biological process.

In RBC2 cells, 20 of the 37 DEMs had |log_2_FC| >1.0, and target predictions for these miRNAs averaged 1800.1 targets in 830.9 genes (Supplementary Table S12). Among the 17 upregulated DEMs, the genes with the highest target scores included *SCAP* (SREBF chaperone), *KAT14* (lysine acetyltransferase 14), *ATP10A* (ATPase phospholipid transporting 10A (putative)), *CSRNP1* (cysteine- and serine-rich nuclear protein 1), *PCSK7* (proprotein convertase subtilisin/kexin type 7), *ABCC2* (ATP-binding cassette subfamily C member 2), *CACNA1E* (calcium voltage-gated channel subunit alpha 1E), *TIMM44* (translocase of inner mitochondrial membrane 44), *OSBP2* (oxysterol binding protein 2), *ACSL6* (acyl-CoA synthetase long-chain family member 6), *TMEM132A* (transmembrane protein 132A), BCL2L1 (BCL2-like 1), *ACTN1* (actinin alpha 1), and the uncharacterized locus *LOC104914368*. The significant enrichment for the target genes include the biological process of multicellular organismal-level homeostasis (GO:0048871; 1.74×; *p* = 5.28E-04) and the molecular function term helicase activity (GO:0004386; 1.85×; *p* = 3.18E-04). For the two downregulated DEM (miR-N56 and miR-N68) genes with the top target alignment scores, we have the following *RBBP8NL* (RBBP8 N-terminal like), *RFX7* (regulatory factor X7), *AANAT* (aralkylamine N-acetyltransferase), *LOC100545461* (antigen-presenting glycoprotein CD1d-like), *LOC100539021* (T-cell surface glycoprotein CD1b-3), and *CADM3* (cell adhesion molecule 3). GO analysis of the suite of predicted targets include the biological function term monoatomic ion transport (GO:0006811; 2.33×; *p* = 6.31E-06) and molecular function terms, such as lipid kinase activity (GO:0001727; 8.42×; *p* = 4.60E-05), active monoatomic ion transmembrane transporter activity (GO:0022853; 3.78×; *p* = 4.40E-05), and active transmembrane transporter activity (GO:0022804; 3.03×; *p* = 2.30E-05).

## Discussion

MicroRNAs are a class of small regulatory RNAs found in almost all animal species that play an important role in controlling the abundance of transcripts in the vertebrate transcriptome (Moran et al., 2017). These short RNA molecules predominantly recognize target sites in the 3′UTRs of mRNAs, typically leading to posttranscriptional repression as a means of modulating the gene expression (Simkin et al., 2020). Posttranscriptional downregulation by miRNAs can have a physiological stimulatory effect, as in the example of the Texel sheep breed where a sequence mutation produced an miR-1/206 binding site, leading to a muscle growth phenotype through the suppression of myostatin (Clop et al., 2006). The modulation of gene expression is critical for embryonic development and cell proliferation in poultry, and miRNAs have been reported to play important roles in these processes (Glazov et al., 2008; Hicks et al., 2008; Harding and Velleman, 2016; Velleman and Harding, 2017; Jebessa et al., 2018). The differential expression of miRNAs associated with growth traits (Li et al., 2011; Andreote et al., 2014; Ouyang et al., 2015) has been reported in chickens. In this study, an extensive set of miRNAs was characterized by small RNA sequencing of turkey *p. major* muscle SCs, identifying a total of 353 miRNAs (161 known and 192 novel). The presence of the known miRNA transcripts in the turkey SCs was consistent with the most abundant miRNAs observed in surveys of chicken skeletal muscles (Li et al., 2011; Ouyang et al., 2015; Khatri et al., 2018).

An expression unique to a limited set of tissues is indicative of highly specific miRNA interactions with a small set of target genes (Bassett et al., 2014). The tissue-specific expression of miRNAs may serve to broadly control translation in specific cells or developmental stages and perhaps modulate developmental fluctuation caused by the environment (Li et al., 2009). The expression of miRNAs is often elevated in specific tissues, and there is strong evidence for the action of specific miRNAs in muscle growth and development (Goljanek-Whysall et al., 2012). For example, miR-206 and closely related members of the miR-1 family are specifically expressed in mammalian muscles (Sempere et al., 2004; McCarthy, 2008; Townley-Tilson et al., 2010) and are required for proper morphogenesis during early embryonic development (Kim et al., 2006; O’Rourke et al., 2007; Ma et al., 2015). The expression of this miRNA family may also be muscle specific in poultry (Li et al., 2011).

The expression of miR-206 in mammals and chicken is enhanced by muscle transcription factors MyoD, MYOG, and myocyte enhancer factor-2 (Mef2) (Rao et al., 2006; Sweetman et al., 2008). However, its expression in mammals is inhibited by transforming growth factor-β (TGF-β) (Winbanks et al., 2011). In bovids, the inhibition of miR-206 and miR-1 was found to enhance SC proliferation (Dai et al., 2016). The downregulation of genes targeted by miR-206 is required for the transition of SCs in mice from proliferation to differentiation (Chen et al., 2006; Chen et al., 2010; Dey et al., 2011). The differential expression of miR-206 in turkeys in proliferating SCs and miR-1 isoforms in differentiating SCs suggests a significant response in SC development resulting from a thermal challenge. However, it is important to note that the functionality of this miRNA may be different in poultry. Associations between the miR-206 expression and general growth (Xu et al., 2013) and more defined traits such as birthweight (Jia et al., 2016), embryo myogenesis (Goljanek-Whysall et al., 2014), and muscle growth (Li et al., 2011) have been reported in chicken. However, few studies have characterized gene interactions with this miRNA. Search for target sites for gga-miR-206 in miRDB identified 675 predicted targets and 356 transcripts with conserved sites predicted using TargetScan. The comparison of these chicken targets with the 529 genes predicted to be targeted by miRanda in turkey found only 12 genes common to all three groups including *ADPGK* (ATP-dependent glucokinase), *COL19A1* (collagen type XIX alpha 1 chain), *FAM91A1* (family with sequence similarity 91 member A1), *KTN1* (kinesin receptor), *MEIS1* (Meis homeobox 1), *NET1* (neuroepithelial cell-transforming 1), *RAPGEF2* (rap guanine nucleotide exchange factor 2), *RNF111* (ring finger protein 111), *SMG7* (nonsense-mediated mRNA decay factor), *TNPO1* (transportin-1), *TRIM2* (tripartite motif-containing 2), and *ZNF827* (zinc finger protein 827), which have various predicted cellular processes but without any notable ties to the SC function or muscle development.

Analysis of miRNAs in turkey SCs found a significant differential expression of known and novel miRNAs, both between genetic lines (RBC2 and NCT) and in response to a thermal challenge. The larger variance component attributed to temperature treatment is expected as a level of physiological response common between the genetic lines and unchanged by selection would be hypothesized. The greater number of DEMs observed in proliferating and differentiating SCs of the RBC2 line compared to the NCT suggests that miRNA response to heat stress has been altered in birds selected for their modern commercial growth traits. Previous RNA-seq studies of mRNA expression within an identical experimental system suggest that growth selection in turkeys has altered the developmental potential of SCs in commercial birds. In proliferating SCs, a greater number of differentially expressed mRNAs were observed from the growth-selected NCT birds, and a pathway analysis indicated a shift toward early myogenesis (Reed et al., 2022a). In differentiating SCs, cold treatment produced expression changes in genes involved in the regulation of skeletal muscle tissue regeneration and sarcomere organization, whereas heat treatment increased the expression of genes regulating myoblast differentiation and survival, particularly in the NCT line (Reed et al., 2022b).

The function of miRNAs in gene regulation is defined by the gene or a group of genes that they target. Target predictions are important in attributing a functional consequence to miRNA differential expression. However, relying on predictions based on comparative datasets (human or chicken) is necessarily biased and highly sensitive to sequence variation due to the small interacting target sequences of miRNAs. Predictions based on the turkey genome and gene set offer a more reliable prediction and sequences. Target prediction algorithms suggest that many miRNAs may interact with a large group of genes, and this is supported by our target predictions. However, the degree to which prediction algorithms identify false positives is a concern (Pinzón et al., 2017; Fridrich et al., 2019). Therefore, the target and pathway predictions resulting from this study necessitate future validation studies to confirm miRNA-specific targets and their functions. Interestingly, three miRNAs (miR-16, miR-24, and miR-128) predicted in an earlier study (Harding and Velleman, 2016), for interacting with genes essential to the SC function (syndecan-4 and glypican-1), were expressed in the present study but were not included among the DEMs.

The interaction of miRNAs with gene targets is a function of the sequence match and accessibility of the target site, as mediated by the secondary structure of target mRNAs (Kertesz et al., 2007). Target sites for miRNAs are also subjected to variable rates of selection, and the sequence conservation of sites is a useful predictor of functionality (Krek et al., 2005). MicroRNAs appear to be under variable selective pressure ranging from strong selection acting on targets of some miRNAs to weak selection for other miRNAs that have many targets (Simkin et al., 2020). While some miRNAs and their targets are highly conserved (Chen and Rajewsky, 2006), others are genus- or species-specific (Kozomara and Griffiths-Jones, 2014). Comparative studies have shown that ancient miRNAs, those highly conserved among divergent taxa, are under stronger selection and are more broadly expressed (Simkin et al., 2020).

Studies have demonstrated that the thermal challenge affects the growth and subsequent structure of poultry breast muscles (Halevy et al., 2001; Piestun et al., 2017; Patael et al., 2019) with downstream effects on the meat quality. A thermal challenge has significant effects on SC proliferation, differentiation, and adipogenic potential with a differential impact on growth-selected lines of turkeys (Clark et al., 2016; Reed et al., 2017a; Reed et al., 2017b; Xu et al., 2021a; Xu et al., 2021b; Reed et al., 2022a; Reed et al., 2022b). The activation and proliferation of SCs is modulated by signaling molecules which direct myogenesis through signaling pathways. These processes are modulated by fine tuning gene expression, likely through RNA/RNA interactions, such as those involving miRNAs. In addition to miRNAs, we also characterize the expression of circular RNAs (circRNAs) in this same experimental system. CircRNAs are novel, single-stranded RNAs that are generated through the splicing of exonic/intronic sequences and are hypothesized to act as miRNA sinks (Wilusz, 2018).

The identification of non-coding RNA molecules provides further insight into the biological response to a thermal challenge and how selection for growth and increased muscle mass has altered this response. Our analyses identified a large number of genes and gene pathways potentially targeted by miRNAs in the turkey SCs available for future studies. The DEMs identified in this study of turkey SCs appear to be related to processes of muscle growth and development similar to their mammalian counterparts, and GO analysis suggests that the differential expression of miRNAs during a thermal challenge significantly affects cellular proliferation and differentiation. We caution that, to date, few studies have directly confirmed molecular miRNA/mRNA interactions in poultry (Velleman and Harding, 2017; Wu et al., 2019; Zhang et al., 2022), and most of the predicted gene interactions are currently based on the assumption that these RNA interactions in bird cells are similar to those observed in mammals (Goljanek-Whysall et al., 2012). There is, however, reason to assume that homologous miRNA:mRNA interactions do exist as target sites for miRNAs are amongst the most highly conserved motifs within mRNA 3′UTRs (Simkin et al., 2020).

This study identified miRNAs expressed in turkey muscle SCs, characterized their differential expression, and predicted important miRNA:mRNA interactions in turkey skeletal muscle SCs. Target gene predictions and Gene Ontology analysis suggest that the differential expression of miRNAs during a thermal challenge could significantly affect SC proliferation and differentiation. The distribution of DEMs suggests that selection for commercial production traits has altered the miRNA expression, providing new hypotheses for future research.

## Data Availability

The datasets presented in this study can be found in online repositories. The names of the repository/repositories and accession number(s) can be found at: https://www.ncbi.nlm.nih.gov/; PRJNA842679.

## References

[B1] Al-Zghoul M. B. Sukker H. Ababneh M. M. (2019). Effect of thermal manipulation of broilers embryos on the response to heat-induced oxidative stress. Poult. Sci. 98, 991–1001. 10.3382/ps/pey379 30137537

[B2] Andreote A. P. Rosario M. F. Ledur M. C. Jorge E. C. Sonstegard T. S. Matukumalli L. (2014). Identification and characterization of microRNAs expressed in chicken skeletal muscle. Genet. Mol. Res. 13, 1465–1479. 10.4238/2014.March.6.5 24634245

[B3] Ason B. Darnell D. K. Wittbrodt B. Berezikov E. Kloosterman W. P. Wittbrodt J. (2006). Differences in vertebrate microRNA expression. Proc. Natl. Acad. Sci. U. S. A. 103, 14385–14389. 10.1073/pnas.0603529103 16983084 PMC1599972

[B4] Barnes N. E. Strasburg G. M. Velleman S. G. Reed K. M. (2019). Thermal challenge alters the transcriptional profile of the breast muscle in turkey poults. Poult. Sci. 98, 74–91. 10.3382/ps/pey401 30239949

[B5] Bassett A. R. Azzam G. Wheatley L. Tibbit C. Rajakumar T. McGowan S. (2014). Understanding functional miRNA-target interactions *in vivo* by site-specific genome engineering. Nat. Commun. 5, 4640. 10.1038/ncomms5640 25135198 PMC4143950

[B6] Chen J. F. Mandel E. M. Thomson J. M. Wu Q. Callis T. E. Hammond S. M. (2006). The role of microRNA-1 and microRNA-133 in skeletal muscle proliferation and differentiation. Nat. Genet. 38, 228–233. 10.1038/ng1725 16380711 PMC2538576

[B7] Chen J. F. Tao Y. Li J. Deng Z. Yan Z. Xiao X. (2010). microRNA-1 and microRNA-206 regulate skeletal muscle satellite cell proliferation and differentiation by repressing Pax7. J. Cell Biol. 190, 867–879. 10.1083/jcb.200911036 20819939 PMC2935565

[B8] Chen K. Rajewsky N. (2006). Deep conservation of microRNA-target relationships and 3’UTR motifs in vertebrates, flies, and nematodes. Cold Spring Harb. Symp. Quant. Biol. 71, 149–156. 10.1101/sqb.2006.71.039 17381291

[B9] Chen Y. Lun A. T. L. Smyth G. K. (2016). From reads to genes to pathways: differential expression analysis of RNA-Seq experiments using Rsubread and the edgeR quasi-likelihood pipeline. F1000Research 5, 1438. 10.12688/f1000research.8987.2 27508061 PMC4934518

[B10] Chen Y. Wang X. (2020). miRDB: an online database for prediction of functional microRNA targets. Nucleic Acids Res. 48 (D1), D127-D131–D131. 10.1093/nar/gkz757 31504780 PMC6943051

[B11] Clark D. L. Coy C. S. Strasburg G. M. Reed K. M. Velleman S. G. (2016). Temperature effect on proliferation and differentiation of satellite cells from turkeys with different growth rates. Poult. Sci. 95, 934–947. 10.3382/ps/pev437 26769270

[B12] Clop A. Marcq F. Takeda H. Pirottin D. Tordoir X. Bibé B. (2006). A mutation creating a potential illegitimate microRNA target site in the myostatin gene affects muscularity in sheep. Nat. Genet. 38, 813–818. 10.1038/ng1810 16751773

[B13] Conway J. R. Lex A. Gehlenborg N. (2017). UpSetR: an R package for the visualization of intersecting sets and their properties. Bioinformatics 33, 2938–2940. 10.1093/bioinformatics/btx364 28645171 PMC5870712

[B14] Dai Y. Wang Y. M. Zhang W. R. Liu X. F. Li X. Ding X. B. (2016). The role of microRNA-1 and microRNA-206 in the proliferation and differentiation of bovine skeletal muscle satellite cells. Vitro Cell Dev. Biol. Anim. 52, 27–34. 10.1007/s11626-015-9953-4 26424132

[B15] Danecek P. Bonfield J. K. Liddle J. Marshall J. Ohan V. Pollard M. O. (2021). Twelve years of SAMtools and BCFtools. GigaScience 10 (2), giab008. 10.1093/gigascience/giab008 33590861 PMC7931819

[B16] Dey B. K. Gagan J. Dutta A. (2011). miR-206 and -486 induce myoblast differentiation by downregulating Pax7. Mol. Cell Biol. 31, 203–214. 10.1128/MCB.01009-10 21041476 PMC3019853

[B17] Enright A. J. John B. Gaul U. Tuschl T. Sander C. Marks D. S. (2003). MicroRNA targets in Drosophila. Genome Biol. 5, R1. 10.1186/gb-2003-5-1-r1 14709173 PMC395733

[B18] Ewels P. Magnusson M. Lundin S. Käller M. (2016). MultiQC: summarize analysis results for multiple tools and samples in a single report. Bioinformatics 32, 3047–3048. 10.1093/bioinformatics/btw354 27312411 PMC5039924

[B19] Fridrich A. Hazan Y. Moran Y. (2019). Too many false targets for microRNAs: challenges and pitfalls in prediction of miRNA targets and their gene ontology in model and non-model organisms. Bioessays 41, e1800169. 10.1002/bies.201800169 30919506 PMC6701991

[B20] Friedländer M. R. Mackowiak S. D. Li N. Chen W. Rajewsky N. (2012). miRDeep2 accurately identifies known and hundreds of novel microRNA genes in seven animal clades. Nucleic Acids Res. 40, 37–52. 10.1093/nar/gkr688 21911355 PMC3245920

[B21] Fu S. Zhao Y. Li Y. Li G. Chen Y. Li Z. (2018). Characterization of miRNA transcriptome profiles related to breast muscle development and intramuscular fat deposition in chickens. J. Cell Biochem. 119, 7063–7079. 10.1002/jcb.27024 29737555

[B22] Glazov E. A. Cottee P. A. Barris W. C. Moore R. J. Dalrymple B. P. Tizard M. L. (2008). A microRNA catalog of the developing chicken embryo identified by a deep sequencing approach. Genome Res. 18, 957–964. 10.1101/gr.074740.107 18469162 PMC2413163

[B23] Goljanek-Whysall K. Mok G. F. Fahad Alrefaei A. Kennerley N. Wheeler G. N. Münsterberg A. (2014). myomiR-dependent switching of BAF60 variant incorporation into Brg1 chromatin remodeling complexes during embryo myogenesis. Development 141, 3378–3387. 10.1242/dev.108787 25078649 PMC4199139

[B24] Goljanek-Whysall K. Sweetman D. Münsterberg A. E. (2012). microRNAs in skeletal muscle differentiation and disease. Clin. Sci. (Lond). 123, 611–625. 10.1042/CS20110634 22888971

[B25] Griffiths‐Jones S. (2004). The microRNA registry. Nucl. Acids Res. 32, D109–D111. 10.1093/nar/gkh023 14681370 PMC308757

[B26] Griffiths-Jones S. Saini H. K. Van Dongen S. Enright A. J. (2008). miRBase: tools for microRNA genomics. Nucleic Acids Res. 36, D154–D158. 10.1093/nar/gkm952 17991681 PMC2238936

[B27] Halevy O. Geyra A. Barak M. Uni Z. Sklan D. (2000). Early posthatch starvation decreases satellite cell proliferation and skeletal muscle growth in chicks. J. Nutr. 130, 858–864. 10.1093/jn/130.4.858 10736342

[B28] Halevy O. Krispin A. Leshem Y. McMurtry J. P. Yahav S. (2001). Early-age heat exposure affects skeletal muscle satellite cell proliferation and differentiation in chicks. Am. J. Physiol-Reg I. 281, R302–R309. 10.1152/ajpregu.2001.281.1.R302 11404306

[B29] Harding R. L. Velleman S. C. (2016). MicroRNA regulation of myogenic satellite cell proliferation and differentiation. Mol. Cell Biochem. 412, 181–195. 10.1007/s11010-015-2625-6 26715133

[B30] Henrikson Z. A. Vermette C. J. Schwean-Lardner K. Crowe T. G. (2018). Effects of cold exposure on physiology, meat quality, and behavior of turkey hens and toms crated at transport density. Poult. Sci. 97, 347–357. 10.3382/ps/pex227 29244085

[B31] Hicks J. A. Tembhurne P. Liu H. C. (2008). MicroRNA expression in chicken embryos. Poult. Sci. 87, 2335–2343. 10.3382/ps.2008-00114 18931185

[B32] Hoffman G. E. Schadt E. E. (2016). variancePartition: interpreting drivers of variation in complex gene expression studies. BMC Bioinforma. 17, 483. 10.1186/s12859-016-1323-z PMC5123296 27884101

[B33] Hulsen T. de Vlieg J. Alkema W. (2008). BioVenn – a web application for the comparison and visualization of biological lists using area-proportional Venn diagrams. BMC Genomics 9, 488. 10.1186/1471-2164-9-488 18925949 PMC2584113

[B34] Jebessa E. Ouyang H. Abdalla B. A. Li Z. Abdullahi A. Y. Liu Q. (2018). Characterization of miRNA and their target gene during chicken embryo skeletal muscle development. Oncotarget 9, 17309–17324. 10.18632/oncotarget.22457 29707110 PMC5915118

[B35] Jia X. Lin H. Abdalla B. A. Nie Q. (2016). Characterization of miR-206 promoter and its association with birthweight in chicken. Int. J. Mol. Sci. 17, 559. 10.3390/ijms17040559 27089330 PMC4849015

[B36] Kertesz M. Iovino N. Unnerstall U. Gaul U. Segal E. (2007). The role of site accessibility in microRNA target recognition. Nat. Genet. 39, 1278–1284. 10.1038/ng2135 17893677

[B37] Khatri B. Seo D. Shouse S. Pan J. H. Hudson N. J. Kim J. K. (2018). MicroRNA profiling associated with muscle growth in modern broilers compared to an unselected chicken breed. BMC Genomics 19, 683. 10.1186/s12864-018-5061-7 30223794 PMC6142689

[B38] Kim H. K. Lee Y. S. Sivaprasad U. Malhotra A. Dutta A. (2006). Muscle-specific microRNA miR-206 promotes muscle differentiation. J. Cell Biol. 174, 677–687. 10.1083/jcb.200603008 16923828 PMC2064311

[B39] Kloosterman W. P. Plasterk R. H. (2006). The diverse functions of microRNAs in animal development and disease. Dev. Cell. 11, 441–450. 10.1016/j.devcel.2006.09.009 17011485

[B40] Kozomara A. Griffiths-Jones S. (2014). miRBase: annotating high confidence microRNAs using deep sequencing data. Nucl. Acids Res. 42, D68–D73. 10.1093/nar/gkt1181 24275495 PMC3965103

[B41] Krek A. Grün D. Poy M. N. Wolf R. Rosenberg L. Epstein E. J. (2005). Combinatorial microRNA target predictions. Nat. Genet. 37, 495–500. 10.1038/ng1536 15806104

[B42] Lang L. Xu B. Li S. Z. Guo W. Yuan J. Zang S. (2019). Rno-miR-425-5p targets the DLST and SLC16A1 genes to reduce liver damage caused by excessive energy mobilization under cold stress. J. Anim. Physiol. Anim. Nutr. Berl. 103, 1251–1262. 10.1111/jpn.13100 31087708

[B43] Langmead B. Trapnell C. Pop M. Salzberg S. L. (2009). Ultrafast and memory-efficient alignment of short DNA sequences to the human genome. Genome Biol. 10, R25. 10.1186/gb-2009-10-3-r25 19261174 PMC2690996

[B44] Li T. Wu R. Zhang Y. Zhu D. (2011). A systematic analysis of the skeletal muscle miRNA transcriptome of chicken varieties with divergent skeletal muscle growth identifies novel miRNAs and differentially expressed miRNAs. BMC Genomics 12, 186. 10.1186/1471-2164-12-186 21486491 PMC3107184

[B45] Li X. Cassidy J. J. Reinke C. A. Fischboeck S. Carthew R. W. (2009). A microRNA imparts robustness against environmental fluctuation during development. Cell 137, 273–282. 10.1016/j.cell.2009.01.058 19379693 PMC2674871

[B46] Li Z. Yaou X. Yaqiu L. (2018). Transcriptome analyses reveal genes of alternative splicing associated with muscle development in chickens. Gene 676, 146–155. 10.1016/j.gene.2018.07.027 30010040

[B47] Liao Y. Smyth G. K. Shi W. (2014). featureCounts: an efficient general purpose program for assigning sequence reads to genomic features. Bioinformatics 30, 923–930. 10.1093/bioinformatics/btt656 24227677

[B48] Liu J. Li F. Hu X. Cao D. Liu W. Han H. (2021). Deciphering the miRNA transcriptome of breast muscle from the embryonic to post-hatching periods in chickens. BMC Genomics 22, 64. 10.1186/s12864-021-07374-y 33468053 PMC7816426

[B49] Loyau T. Metayer-Coustard S. Berri C. Crochet S. Cailleau-Audouin E. Sannier M. (2014). Thermal manipulation during embryogenesis has long-term effects on muscle and liver metabolism in fast-growing chickens. PLoS ONE 9, e105339. 10.1371/journal.pone.0105339 25180913 PMC4152147

[B50] Ma G. Wang Y. Li Y. Cui L. Zhao Y. Zhao B. (2015). MiR-206, a key modulator of skeletal muscle development and disease. Int. J. Biol. Sci. 11, 345–352. 10.7150/ijbs.10921 25678853 PMC4323374

[B51] Martin M. (2011). Cutadapt removes adapter sequences from high-throughput sequencing reads. EMBnet.J. 17, 10–12. 10.14806/ej.17.1.200

[B52] McCarthy J. J. (2008). MicroRNA-206: the skeletal muscle-specific myomiR. Biochim. Biophys. Acta 1779, 682–691. 10.1016/j.bbagrm.2008.03.001 18381085 PMC2656394

[B53] McGeary S. E. Lin K. S. Shi C. Y. Pham T. Bisaria N. Kelley G. M. (2019). The biochemical basis of microRNA targeting efficacy. Science 366, eaav1741. 10.1126/science.aav1741 31806698 PMC7051167

[B54] Moran Y. Agron M. Praher D. Technau U. (2017). The evolutionary origin of plant and animal microRNAs. Nat. Ecol. Evol. 1, 27. 10.1038/s41559-016-0027 28529980 PMC5435108

[B55] Mozdziak P. E. Walsh T. J. McCoy D. W. (2002). The effect of early posthatch nutrition on satellite cell mitotic activity. Poult. Sci. 81, 1703–1708. 10.1093/ps/81.11.1703 12455598

[B56] Nawab A. Ibtisham F. Li G. Kieser B. Wu J. Liu W. (2018). Heat stress in poultry production: mitigation strategies to overcome the future challenges facing the global poultry industry. J. Therm. Biol. 78, 131–139. 10.1016/j.jtherbio.2018.08.010 30509629

[B57] Nestor K. E. McCartney M. G. Bachev N. (1969). Relative contributions of genetics and environment to turkey improvement. Poult. Sci. 48, 1944–1949. 10.3382/ps.0481944 5373754

[B58] O'Rourke J. R. Georges S. A. Seay H. R. Tapscott S. J. McManus M. T. Goldhamer D. J. (2007). Essential role for Dicer during skeletal muscle development. Dev. Biol. 311, 359–368. 10.1016/j.ydbio.2007.08.032 17936265 PMC2753295

[B59] Ouchi Y. Chowdhury V. S. Cockrem J. F. Bungo T. (2021). Effects of thermal conditioning on changes in hepatic and muscular tissue associated with reduced heat production and body temperature in young chickens. Front. Vet. Sci. 7, 610319. 10.3389/fvets.2020.610319 33537354 PMC7847892

[B60] Ouyang H. He X. Li G. Xu H. Jia X. Nie Q. (2015). Deep sequencing analysis of miRNA expression in breast muscle of fast-growing and slow-growing broilers. Int. J. Mol. Sci. 16, 16242–16262. 10.3390/ijms160716242 26193261 PMC4519947

[B61] Patael T. Piestun Y. Soffer A. Mordechay S. Yahav S. Velleman S. G. (2019). Early posthatch thermal stress causes long-term adverse effects on pectoralis muscle development in broilers. Poult. Sci. 98, 3268–3277. 10.3382/ps/pez123 31041445

[B62] Piestun Y. Druyan S. Brake J. Yahav S. (2013). Thermal manipulations during broiler incubation alter performance of broilers to 70 days of age. Poult. Sci. 92, 1155–1163. 10.3382/ps.2012-02609 23571323

[B63] Piestun Y. Patael T. Yahav S. Velleman S. G. Halevy O. (2017). Early posthatch thermal stress affects breast muscle development and satellite cell growth and characteristics in broilers. Poult. Sci. 96, 2877–2888. 10.3382/ps/pex065 28444312

[B64] Pinzón N. Li B. Martinez L. Sergeeva A. Presumey J. Apparailly F. (2017). microRNA target prediction programs predict many false positives. Genome Res. 27, 234–245. 10.1101/gr.205146.116 28148562 PMC5287229

[B65] Quast C. Pruesse E. Yilmaz P. Gerken J. Schweer T. Yarza P. (2013). The SILVA ribosomal RNA gene database project: improved data processing and web-based tools. Nucleic Acids Res. 41, D590–D596. 10.1093/nar/gks1219 23193283 PMC3531112

[B66] Rao P. K. Kumar R. M. Farkhondeh M. Baskerville S. Lodish H. F. (2006). Myogenic factors that regulate expression of muscle-specific microRNAs. Proc. Natl. Acad. Sci. U. S. A. 103, 8721–8726. 10.1073/pnas.0602831103 16731620 PMC1482645

[B67] Raza S. H. A. Abdelnour S. A. Dhshan A. I. M. Hassanin A. A. Noreldin A. E. Albadrani G. M. (2021). Potential role of specific microRNAs in the regulation of thermal stress response in livestock. J. Therm. Biol. 96, 102859. 10.1016/j.jtherbio.2021.102859 33627286

[B68] R Core Team (2022). R: a language and environment for statistical computing. Vienna, Austria: R Foundation for Statistical Computing. Available at: https://www.R-project.org/.

[B69] Reed K. M. Mendoza K. M. Abrahante J. E. Barnes N. E. Velleman S. G. Strasburg G. M. (2017a). Response of turkey muscle satellite cells to thermal challenge. I. Transcriptome effects in proliferating cells. BMC Genomics 18, 352. 10.1186/s12864-017-3740-4 28477619 PMC5420122

[B70] Reed K. M. Mendoza K. M. Strasburg G. M. Velleman S. G. (2017b). Response of turkey muscle satellite cells to thermal challenge. II. Transcriptome effects in differentiating cells. Front. Physiol. 8, 948. 10.3389/fphys.2017.00948 29249977 PMC5714890

[B71] Reed K. M. Mendoza K. M. Strasburg G. M. Velleman S. G. (2022a). Transcriptome response of proliferating muscle satellite cells to thermal challenge in commercial turkey. Front. Physiol. 13, 970243. 10.3389/fphys.2022.970243 36091406 PMC9452691

[B72] Reed K. M. Mendoza K. M. Strasburg G. M. Velleman S. G. (2022b). Transcriptome response of differentiating muscle satellite cells to thermal challenge in commercial turkey. Genes 13, 1857. 10.3390/genes13101857 36292741 PMC9601516

[B73] Robinson M. D. McCarthy D. J. Smyth G. K. (2010). edgeR: a Bioconductor package for differential expression analysis of digital gene expression data. Bioinformatics 26, 139–140. 10.1093/bioinformatics/btp616 19910308 PMC2796818

[B74] Saliminejad K. Khorram Khorshid H. R. Soleymani Fard S. Ghaffari S. H. (2019). An overview of microRNAs: biology, functions, therapeutics, and analysis methods. J. Cell Physiol. 234, 5451–5465. 10.1002/jcp.27486 30471116

[B75] Sempere L. F. Freemantle S. Pitha-Rowe I. Moss E. Dmitrovsky E. Ambros V. (2004). Expression profiling of mammalian microRNAs uncovers a subset of brain-expressed microRNAs with possible roles in murine and human neuronal differentiation. Genome Biol. 5, R13. 10.1186/gb-2004-5-3-r13 15003116 PMC395763

[B76] Sengar G. S. Deb R. Singh U. Raja T. V. Kant R. Sajjanar B. (2018). Differential expression of microRNAs associated with thermal stress in Frieswal (*Bos taurus* x *Bos indicus*) crossbred dairy cattle. Cell Stress Chap 23, 155–170. 10.1007/s12192-017-0833-6 PMC5741590 28776223

[B77] Shu J. Liu Y. Shan Y. Ji G. Ju X. Tu Y. (2021). Deep sequencing microRNA profiles associated with wooden breast in commercial broilers. Poult. Sci. 100, 101496. 10.1016/j.psj.2021.101496 34695627 PMC8555438

[B78] Simkin A. Geissler R. McIntyre A. B. R. Grimson A. (2020). Evolutionary dynamics of microRNA target sites across vertebrate evolution. PLoS Genet. 16, e1008285. 10.1371/journal.pgen.1008285 32012152 PMC7018135

[B79] Sweetman D. Goljanek K. Rathjen T. Oustanina S. Braun T. Dalmay T. (2008). Specific requirements of MRFs for the expression of muscle specific microRNAs, miR-1, miR-206 and miR-133. Dev. Biol. 321, 491–499. 10.1016/j.ydbio.2008.06.019 18619954

[B80] Tange O. (2018). GNU parallel 2018. Lulu.com, 112. 10.5281/zenodo.1146014

[B81] Townley-Tilson W. H. Callis T. E. Wang D. (2010). MicroRNAs 1, 133, and 206: critical factors of skeletal and cardiac muscle development, function, and disease. Int. J. Biochem. Cell Biol. 42, 1252–1255. 10.1016/j.biocel.2009.03.002 20619221 PMC2904322

[B82] Velleman S. G. Harding R. L. (2017). Regulation of turkey myogenic satellite cell migration by microRNAs miR-128 and miR-24. Poult. Sci. 96, 1910–1917. 10.3382/ps/pew434 27920196

[B83] Wienholds E. Kloosterman W. P. Miska E. Alvarez-Saavedra E. Berezikov E. de Bruijn E. (2005). MicroRNA expression in zebrafish embryonic development. Science 309, 310–311. 10.1126/science.1114519 15919954

[B84] Wilson H. R. Wilcox C. J. Voitle R. A. Baird C. D. Dorminey R. W. (1975). Characteristics of White Leghorn chickens selected for heat tolerance. Poult. Sci. 54, 126–130. 10.3382/ps.0540126 1135123

[B85] Wilusz J. E. (2018). A 360˚ view of circular RNAs: from biogenesis to functions. Wiley Interdiscip. Rev. RNA 9, e1478. 10.1002/wrna.1478 29655315 PMC6002912

[B86] Winbanks C. E. Wang B. Beyer C. Koh P. White L. Kantharidis P. (2011). TGF-beta regulates miR-206 and miR-29 to control myogenic differentiation through regulation of HDAC4. J. Biol. Chem. 286, 13805–13814. 10.1074/jbc.M110.192625 21324893 PMC3077581

[B87] Wu N. Gu T. Lu L. Cao Z. Song Q. Wang Z. (2019). Roles of miRNA-1 and miRNA-133 in the proliferation and differentiation of myoblasts in duck skeletal muscle. J. Cell Physiol. 234, 3490–3499. 10.1002/jcp.26857 30471101

[B88] Xu J. Strasburg G. M. Reed K. M. Velleman S. G. (2021a). Response of turkey pectoralis major muscle satellite cells to hot and cold thermal stress: effect of growth selection on satellite cell proliferation and differentiation. Comp. Biochem. Physiol. A 252, 110823. 10.1016/j.cbpa.2020.110823 33148517

[B89] Xu J. Strasburg G. M. Reed K. M. Velleman S. G. (2021b). Effect of temperature and selection for growth on intracellular lipid accumulation and adipogenic gene expression in turkey pectoralis major muscle satellite cells. Front. Physiol. 12, 667814. 10.3389/fphys.2021.667814 34140894 PMC8204085

[B90] Xu Z. Nie Q. Zhang X. (2013). Overview of genomic insights into chicken growth traits based on genome-wide association study and microRNA regulation. Curr. Genomics 14, 137–146. 10.2174/1389202911314020006 24082823 PMC3637678

[B91] Zhang G. Zhang X. Zhou K. Ling X. Zhang J. Wu P. (2022). miRNA-10a-5p targeting the BCL6 gene regulates proliferation, differentiation and apoptosis of chicken myoblasts. Int. J. Mol. Sci. 23, 9545. 10.3390/ijms23179545 36076940 PMC9455618

